# Trials Methodology Research: what is it and why should India invest in it?

**DOI:** 10.1016/j.lansea.2024.100360

**Published:** 2024-02-02

**Authors:** Sangeetha Paramasivan, Anant Bhan, Rashmi Rodrigues, Usha Menon

**Affiliations:** a Population Health Sciences, Bristol Medical School, University of Bristol, Bristol, United Kingdom; b Bhopal Hub, Sangath, India; c Centre for Ethics, Yenepoya (Deemed to be University), Mangaluru, Karnataka, India; d Community Health, St. John's Medical College, St. John's National Academy of Health Sciences, Bengaluru, Karnataka, India; e MRC Clinical Trials Unit, University College London, Institute of Clinical Trials and Methodology, London, United Kingdom

**Keywords:** Clinical trials, Trials methodology research, Randomised controlled trials, India

## Abstract

This Viewpoint presents an overview of trials methodology research (TMR) and the case for investing in TMR in India. Randomised controlled trials and other types of clinical research inform evidence-based medicine, but this endeavour is dependent on the quality of such research. TMR is aimed at improving the way in which clinical trials are designed, conducted, analysed, and reported. The evolution of TMR in countries like the UK has been nurtured through dedicated funding support. Similar funding opportunities for TMR in India will help optimise the ethical and methodological rigour of the growing number of trials conducted in India. Such funding could help initiate an interdisciplinary network of key stakeholders in India to lead on TMR priority-setting exercises so that methodological questions of relevance to India are addressed. The establishment of trials methodology hubs will enhance initiatives such as the disease-specific clinical trials networks being set up as part of the National Biopharma Mission in India. We posit that promoting and establishing TMR as a distinct field of study in India will ensure the improvement of our health research ecosystem and call on national and international funding bodies to initiate consultation, consensus building and ringfenced funding for TMR in India.

## Introduction

High-quality health research is imperative for robust clinical decision-making as well as formulating and reforming public health policies. Randomised controlled trials (RCTs) are unique for many reasons—they have a pivotal role in evidence-based medicine^1^ and have exclusive design aspects that make them particularly challenging to conduct in comparison to other research designs.^2^^,^^3^ RCTs are underpinned by complex concepts such as clinical equipoise^4^ and randomisation.^5^ They also require sample sizes that are sufficiently large to detect clinically significant differences between the treatments being compared^2^ and the selection of outcome measures that are important to patients and clinicians.^6^ While the theoretical principles and bioethical concepts underpinning RCTs are universal^5^ (e.g. avoiding bias and confounding factors, ensuring appropriate sample size and randomisation methods, and protecting patients’ autonomy and safety), how the concepts are operationalised across various settings is different. The manner in which RCTs are operationalised is as integral to their outcome as the intervention being evaluated and the finer design details. There is a need for researchers to continually evaluate how RCTs are conducted in their settings and suggest improvements. Trials methodology research (TMR) increasingly plays a crucial role in such an endeavour. In this Viewpoint, we outline what TMR is, invite further discussions on its scope for India, consider why it is an important area for national and international funders and what can be achieved with such funding.

## What is trials methodology research (TMR)?

It is research aimed at improving how clinical trials are designed, conducted, analysed, and reported. TMR includes, in addition to RCTs, qualitative research and a variety of mixed methods.^7^^,^^8^ The remit is extensive and continually evolving. Examples include (i) evaluating the use of routinely collected patient data for outcomes^9^^,^^10^ and the development of reporting guidelines for such RCTs^11^^,^^12^; (ii) optimising the recruitment and informed consent process by exploring how to improve the ways in which patients are identified, screened, approached and invited to participate in trials^13^; and (iii) developing novel methods, such as adaptive trial designs, that are flexible and allow modifications within pre-specified rules.^14^ Patients' and carers’ perspectives and lived experiences are increasingly acknowledged as essential to determining outcome measures in RCTs.^15^^,^^16^ This has resulted in patient and public involvement (PPI)^7^^,^^8^ becoming an integral part of TMR.^17^^,^^18^

In the UK, TMR has evolved as a distinct field over the past couple of decades, nurtured by funding from the Medical Research Council (MRC). This allowed the establishment of methodological hubs in five universities in the UK, with each focussing on different aspects of trials methodology.^19^ This initiative evolved into the MRC-NIHR (National Institute for Health Research) Trials Methodology Research Partnership (TMRP) that included a Global Health working group, which launched trials methodology pump-priming grants for low-income and middle-income countries (LMICs). In the first round, seven projects were awarded funding (out of over 270 applications received worldwide), with one based in India.^20^

There may be some confusion with another similar phrase to TMR–‘Research on Research,’ (RoR)^21^ sometimes called meta-research, meta-science and science of science, and supported by two major UK funding bodies—Wellcome Trust^22^ and NIHR,^23^ which have both established RoR programmes^24^ and institutes.^25^ RoR has a wider canvas than TMR and focuses more on the structural, organisational and operational aspects of research (e.g. scrutinising grant application criteria and its influence on inequities in research funding.^25^

## What could TMR mean in India?

The regulatory reforms introduced since 2013,^26^ including the New Drugs and Clinical Trials Rules of 2019,^27^ have helped streamline the approval processes required for conducting clinical trials in India. The reported increase in the number of pharmaceutical trials in India over recent years^28^ (after a lull following legal action for allegations of unethical trial conduct^29^) provides a fresh opportunity to ensure that trials are ethically and methodologically rigorous. The latter is a key aim of TMR and will contribute towards stronger health research systems that can bolster public confidence in healthcare and medicine.

In India, within the regulatory framework, the term ‘clinical trials’ is limited to the study of ‘new drugs’ only.^27^ However, the Clinical Trials Registry-India (CTRI)^30^ is broader in its scope and registers trials of any intervention, which includes drugs, surgical procedures, preventive measures, lifestyle modifications, devices, education, or behavioural treatments. Similarly, it would be important for TMR to be carried out on different types of interventional research (medical, surgical, behavioural, and public health or health systems) and observational studies (e.g. cohort studies).

TMR is not new to India and has been undertaken without using similar terminology. Examples include studies that explore willingness to participate in clinical trials,^31^ randomised comparisons of interventions to aid participant comprehension^32^ and public and professional perceptions on biobanking research.^33^^,^^34^ A scoping review of Indian studies on clinical research ethics^35^ found that although a wide range of topics were covered, the focus was on knowledge assessments amongst easily accessible groups such as ethics committee members and healthcare students on topics such as research ethics. Gaps identified, in consultation with key stakeholders from India, included developing a better understanding of the recruitment and informed consent process (e.g. recruiter–participant interaction), developing models of informed consent that are specific to the Indian context and exploring issues such as equity and justice within the context of clinical trials. However, given the above review's^35^ focus on empirical research on the ethics of clinical research in India rather than the broader area of TMR, it is possible that existing TMR studies in India have been missed. A comprehensive scoping review focussed on TMR in India would be a good starting point to identify gaps in TMR that need to be addressed in the Indian context.

Promoting and establishing TMR as a distinct field of study in India (see Box 1 for key recommendations) would help ensure more efficient and safe ways of conducting research that informs national health policies and supports research teams to, in addition to generics production, also focus on the development of new drugs with Indian intellectual property.^36^^,^^37^ For instance, we know little about how consent is obtained for trials within emergency settings in India or how assent is obtained in trials with children as participants.^35^ TMR can help understand and optimise such trial processes, which can inform wider evidence-based guidance on obtaining consent in challenging situations in the Indian context. Ensuring we have established informed consent processes in place will be useful when faced with a rapidly evolving crisis that warrants the development and trialling of new drugs, such as during the recent pandemic. Similarly, TMR can help investigate how to make trials part of routine patient care in a country as diverse as India—a strategy that can help reduce the burden on busy healthcare staff, streamline trial procedures and contribute to improving the cost-efficiency of trials.

Box 1 **Key recommendations to promote and establish Trials Methodology Research (TMR) as a distinct field of study in India.**

• Initiate consultation, consensus building and ringfenced funding streams for TMR in India to help optimise the ethical and methodological rigour of clinical trials in the country.
• Establish an interdisciplinary network of key stakeholders to set the agenda and scope for TMR in India.
• Conduct inclusive and collaborative priority-setting exercises to elicit key trials methodological questions that need to be addressed in the Indian context.
• Fund proposals that address the identified research priorities.
• Establish trials methodology hubs to enhance existing initiatives and networks that are aimed at improving the health research ecosystem in India.

## Is there adequate funding for TMR in India?

TMR often falls through the gaps in funding offered by national and international bodies. TMR is often considered too methodological for priority areas identified within ‘Global Challenges’^38^ and too ‘global health’ or applied for purely methodological funding streams. Apart from the TMRP Global Health pump-priming grant,^20^ there are currently no specific international funding opportunities to conduct methodological research in LMICs such as India. This is reflected in the scoping review mentioned above ^35^ which demonstrated that most Indian studies on clinical research ethics were conducted with limited to no funding.

Establishing trials methodology hubs that are based within research-active institutions in India would be a turning point in ensuring methodological research questions of relevance to the country are initiated and conducted. For instance, most of the clinical research ethics studies in India have used questionnaire surveys of uncertain or inadequate quality to assess participant comprehension and knowledge.^35^ Funded methodological research would enable the development of India-specific culturally relevant knowledge assessment tools. Given the linguistic diversity, they may require validation in multiple languages and could be used to assess informed consent quality across multiple Indian trials or studies.

Trials methodology hubs established within centres of excellence in India would serve to amplify the good methodological practices employed by these centres, while simultaneously reaching out to traditionally less research-active centres. They would complement the disease specific clinical trials networks and the efforts to enhance research ethics capacity^39^ that are being set up as part of the National Biopharma Mission and other initiatives.^40^ None of this would be possible without adequate funding—whether from national or international bodies—and lobbying for this would be a pivotal step in ensuring the improvement of our health research ecosystem in India. A key incentive for funding bodies to prioritise TMR in India is to reduce research waste and ensure better value for money in the delivery of practice changing high-quality and cost-efficient RCTs.

## What could be achieved if there was strategic funding for TMR in India?

Initially, this could provide for a large-scale collaborative and interdisciplinary network of key stakeholders such as patient and public representatives, clinicians, trialists, methodologists (qualitative and quantitative), social scientists, bioethicists, funding body representatives and health activists to set the agenda and scope for TMR in India. The network could lead on a priority-setting exercise^41^ to elicit key methodological questions that need to be addressed for the field, for specific disciplines (such as cancer trials), subject areas (such as ethical aspects of clinical trials or clinical research,^35^ including with marginalised populations) or areas of concern (such as informed consent and monitoring and oversight of study conduct). Funded calls for proposals addressing research priorities could then set the scene for developing the evidence base that will help improve the conduct and efficiency of clinical research studies and trials. Such an approach over nearly two decades has led to significant strides in the field of TMR in the UK.^42^

The network of stakeholders could also consider potential challenges and solutions related to the operationalisation of TMR in India. There is likely to be a need for concerted efforts to publicise the importance and benefits of TMR amongst wider patient, public, clinical and research groups. While the bulk of TMR can be carried out by researchers with existing methodological expertise in qualitative, quantitative, or mixed methods research, there may be some need for re-casting of this expertise. This may mean ensuring that, where necessary, TMR Studies Within a Trial (SWAT)^43^ include a component that is directly applicable to refining the design, conduct or reporting of the particular trial. For example, a pre-RCT qualitative study can help refine or identify additional clinical questions or modify design and conduct so that it is more participant friendly. Similarly, TMR exploring participants’ understanding of trial procedures or randomisation could incorporate a feedback phase within its design^13^ to share the findings with the trial team and incorporate evidence-based solutions to recruitment and informed consent related barriers.

Research priority setting could also help identify areas where there is a need to address known trials methodology issues. For instance, TMR on the reporting of trial results has consistently shown that a large proportion of Indian clinical trials registered on the CTRI remain unpublished,^44^^,^^45^ emphasising the need for regulations to mandate the timely publication of trial results. Along the same vein, an observational TMR study published in 2013 to evaluate the reporting quality of RCTs found that methods reporting was better in the CTRI than in Indian journal publications, with the latter demonstrating suboptimal compliance with Consolidated Standards for the Reporting of Trials (CONSORT) and International Committee of Medical Journal Editors (ICJME) requirements.^46^ Supportive initiatives to help editors of Indian journals to improve the reporting quality of RCTs will help bridge the mismatch in reporting quality between the CTRI protocol registration stage and RCT results publication stage.

The pandemic and the growing number of Indian trials has created an environment where all stakeholders (researchers, funders, policy makers, patients, and public) are keen to explore and optimise how health research is conducted. The emerging field of TMR in India provides that opportunity. It has immense potential to improve how clinical research and in particular trials and large cohort studies are conducted. We urge the funding bodies to lead the way through consultation, consensus building and dedicated funding streams.

## Contributors

SP conceived the idea, discussed it with all authors and wrote the first draft. All authors contributed to the drafting process, and have read and approved the final version of the manuscript. All authors had final responsibility for the decision to submit for publication.

## Declaration of interests

SP was funded by the MRC ConDuCT-II (Medical Research Council, Collaboration and innovation for Difficult and Complex randomised controlled Trials In Invasive procedures) Hub for Trials Methodology Research (MR/K025643/1) at the University of Bristol, when the ideas for this paper were first conceived and discussed. UM receives salary support from MRC CTU at UCL core funding (MR_UU_12,023). UM has research collaborations focused on early detection of cancer with the following industry–Intelligent Lab on Fiber, RNA Guardian, Micronoma, MercyBio Analytics, and academic partners—Cambridge University, QIMR Berghofer Medical Research Institute, Imperial College London, University of Innsbruck, and Harvard, Duke, Oregon Health and Science and Washington Universities, USA. UM had stock ownership (2011–2021) awarded by University College London (UCL) in Abcodia, which held the licence for the Risk of Ovarian Cancer Algorithm (ROCA). We declare no competing interests.
